# Detrimental infant and maternal outcomes of undiagnosed asymptomatic malaria in pregnancy

**DOI:** 10.1371/journal.pmed.1004528

**Published:** 2025-02-24

**Authors:** James G. Beeson, Daniel Herbert Opi

**Affiliations:** 1 Burnet Institute, Melbourne, Victoria, Australia; 2 Monash University, Melbourne, Victoria, Australia; 3 University of Melbourne, Melbourne, Victoria, Australia

## Abstract

In areas with low malaria transmission intensity, most infections during pregnancy are not detected by standard diagnostic tests. New data shows that these missed asymptomatic infections have substantial negative impacts for both the mother and the developing fetus.

Over 40% of the world’s population is at risk of malaria, yet progress in reducing the global burden has stalled since 2015 [1]. Pregnant women, in particular, are highly susceptible to both infection and disease [2]. Malaria during pregnancy can lead to severe outcomes, including maternal anaemia and low infant birth weight, as well as pre-term birth, miscarriage and stillbirth, among others. Most malaria cases are due to the species *Plasmodium falciparum* and *Plasmodium vivax*, and clinical illness occurs during the blood-stage of infection when *Plasmodium* parasites infect and replicate in red blood cells (RBCs). In pregnant women, infected RBCs can accumulate in the placenta, which is a prominent feature of *P. falciparum* rather than *P. vivax* infection, contributing to negative outcomes for both the mother and developing fetus.

Malaria is typically diagnosed by testing peripheral blood using rapid diagnostic tests that can detect parasite proteins, or by microscopic examination of stained blood smears. While these tests generally perform well for diagnosis of symptomatic malaria illness with a high parasite burden, they have much lower sensitivity as screening tests for malaria infection and in asymptomatic infections in which parasitemia is typically low. Furthermore, they are less sensitive for diagnosis of *P. vivax* infections partly due to its different biology and low parasite densities. More sensitive nucleic acid amplification tests, such as PCR, are available, but currently they are only widely used in surveillance studies and research rather than for point-of-care diagnosis and treatment of malaria due to technical and cost limitations. So-called ‘ultra-sensitive’ PCR tests using high-volume concentrated blood samples have been developed that can detect very low parasitemia. Using these more sensitive tests has revealed that a large proportion of *Plasmodium* spp infections are not detected by standard diagnostic tests [3], and these are often referred to as submicroscopic infections. Studies across multiple populations show that a large proportion of *Plasmodium* infections in pregnancy are submicroscopic, low-density infections [4]. While the negative effects of high-density and microscopically detectable infections on pregnancies are well documented, the impact of low and very low submicroscopic infections in pregnancy is less clear, especially in the Asia-Pacific region where *P. vivax* is often the more prevalent species and co-infections of *P. falciparum* and *P. vivax* are common.

A new study of over 4,000 pregnant women now provides important data demonstrating that even submicroscopic infections can have substantial negative impacts for both the fetus and mother [5]. The study was conducted in a setting of low and unstable malaria transmission around the Thailand–Myanmar border, where both *P. falciparum* and *P. vivax* are endemic, and included women attending their first antenatal clinic (median gestational age 16.5 weeks) who were followed during pregnancy. An important aspect to this study was the use of an ultra-sensitive PCR test capable of detecting as few as 22 parasites per mL of blood. The authors found that, at the first antenatal clinical visit, these submicroscopic infections were 4-times more prevalent than microscopically detectable infections. Thus, screening for infection using standard diagnostics would miss most infections in pregnancy, and even a standard PCR test using dried blood spots failed to detect 84% of infections in this study due to insufficient sensitivity. Repeated follow-up testing of samples from women during pregnancy found that most infections did not become microscopically detectable over time, underscoring the limitations of routine diagnostics in detecting these missed infections.

A crucial finding of this study is the clinical sequelae associated with these low-level, asymptomatic infections: submicroscopic infections were associated with substantially lower infant birth weight, and this negative association was observed for both *P. falciparum* and *P. vivax* infections. Low birth weight is a major risk factor for infant death and can have other long-term impacts including impaired growth and development in the child [6]. *P. falciparum*, but not *P. vivax*, infections were also associated with increased risk of maternal anaemia.

The demonstration that most *Plasmodium* infections in pregnancy are not detected by microscopy or standard PCR, and that despite their low parasite burden, these infections have measurable negative impacts on pregnant women and infants, highlights the importance of preventing malaria in pregnancy. Moreover, these findings have important implications for malaria elimination efforts if a large proportion of infections are undetected by conventional diagnostics, and therefore remain untreated. Key questions and challenges remain about how to combat these missed submicroscopic infections. Implementing routine screening using ultra-sensitive PCR, as used in this study, for all pregnant women in endemic areas is not presently feasible due to financial and technical constraints. Ongoing research around new diagnostic technologies may generate highly sensitive diagnostics in the future that are affordable and easily accessible for screening and treatment.

Strengthening and investing in available malaria control and prevention measures in endemic populations remains an essential complement to development of new diagnostic tools. These include strategies to reduce mosquito exposure, such as insecticide-treated bed nets and spraying of residual insecticides; however, their efficacy is variable depending on the endemic setting. Novel approaches for vector control and prevention of exposure is an area of active research [7], and might generate future tools that reduce malaria and the risk for pregnant women. In settings with moderate-to-high transmission, the use of intermittent preventive treatment or chemoprophylaxis in pregnancy is recommended to clear and prevent infections, but it is generally not recommended in areas of low transmission, in part because of a less favourable balance between risks and benefits. Two new vaccines for *P. falciparum* have recently been approved for implementation in young children [8], but their potential for malaria prevention in pregnant women and in regions with low transmission is not known, and they are not effective against *P. vivax*. Ongoing research is focussed on developing vaccines to prevent infection and interrupt transmission of malaria [9]. Targeted and improved control measures and effective vaccines would go a long way in reducing the huge burden of submicroscopic *Plasmodium* infections and the negative impacts during pregnancy.

It remains unclear how submicroscopic *Plasmodium* infections persist and exert their negative maternal and fetal effects during pregnancy. Further research is needed to elucidate their impact and identify strategies for detection and prevention. Parasitemia in peripheral blood may not reflect the total parasite biomass, particularly since *P. falciparum* can sequester in the placenta. Multi-morbidity is common among pregnant women in malaria endemic populations and low- and middle-income settings and these may contribute to negative outcomes. A better understanding of interactions between detectable and submicroscopic malaria infections and maternal co-morbidities may be valuable in preventing harmful effects and optimising pregnancy outcomes.
